# Toward countering muscle and bone loss with spaceflight: GSK3 as a potential target

**DOI:** 10.1016/j.isci.2023.107047

**Published:** 2023-06-08

**Authors:** Ryan W. Baranowski, Jessica L. Braun, Briana L. Hockey, Jenalyn L. Yumol, Mia S. Geromella, Colton J.F. Watson, Nigel Kurgan, Holt N. Messner, Kennedy C. Whitley, Adam J. MacNeil, Guillemette Gauquelin-Koch, Fabrice Bertile, William Gittings, Rene Vandenboom, Wendy E. Ward, Val A. Fajardo

**Affiliations:** 1 Department of Kinesiology, Brock University, St. Catharines, ON, Canada; 2 Centre for Bone and Muscle Health, Brock University, St. Catharines, ON, Canada; 3 Department of Health Sciences, Brock University, St. Catharines, ON, Canada; 4 French Space Agency, Centre National d'Etudes Spatiales (CNES), Paris, France; 5 Hubert Curien Pluridisciplinary Institute (IPHC), CNRS, Strasbourg University, Strasbourg, France

**Keywords:** Musculoskeletal medicine, Space medicine

## Abstract

We examined the effects of ∼30 days of spaceflight on glycogen synthase kinase 3 (GSK3) content and inhibitory serine phosphorylation in murine muscle and bone samples from four separate missions (BION-M1, rodent research [RR]1, RR9, and RR18). Spaceflight reduced GSK3β content across all missions, whereas its serine phosphorylation was elevated with RR18 and BION-M1. The reduction in GSK3β was linked to the reduction in type IIA fibers commonly observed with spaceflight as these fibers are particularly enriched with GSK3. We then tested the effects of inhibiting GSK3 before this fiber type shift, and we demonstrate that muscle-specific *Gsk3* knockdown increased muscle mass, preserved muscle strength, and promoted the oxidative fiber type with Earth-based hindlimb unloading. In bone, GSK3 activation was enhanced after spaceflight; and strikingly, muscle-specific *Gsk3* deletion increased bone mineral density in response to hindlimb unloading. Thus, future studies should test the effects of GSK3 inhibition during spaceflight.

## Highlights

• GSK3β content was reduced (−36%) in murine soleus muscles after ∼30 days of spaceflight
• *Gsk3* muscle knockdown (GSK3^mKD^) preserved muscle strength after hindlimb suspension
• Spaceflight reduced bone mineral density in mice
• GSK3^mKD^ increased FNDC5 expression and tibia bone mineral density

## Graphical abstract

**Figure:**
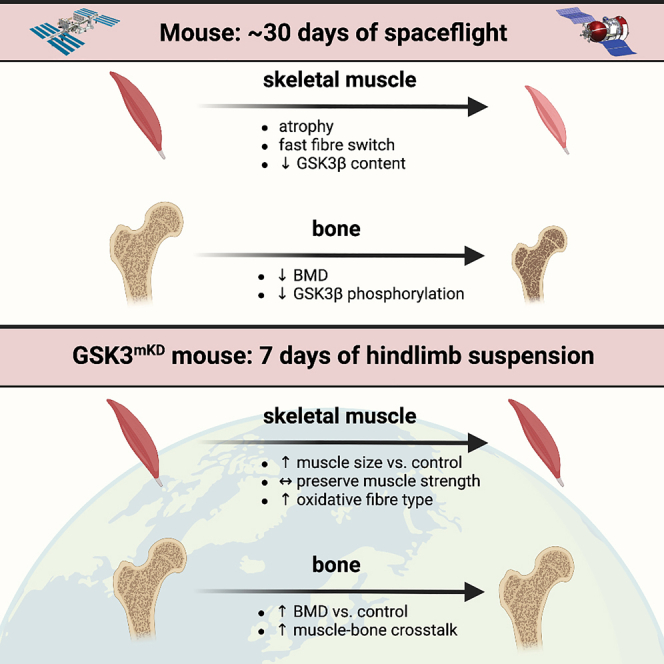

## Introduction

From the first manned flight into space to sending humans to the surface of the moon, we have gained a better appreciation and understanding of the complexities of space travel. Now in the 21st century, current efforts are aimed at a return to the moon, largely in part as preparation for longer duration missions that take humans to Mars for the first time. However, as space exploration advances into new environmental domains, the complications with basic human survival and physiology become even more evident. NASA has identified several hazards of human spaceflight including space radiation, isolation and confinement, distance from earth (and its technological hurdles), exposure to extreme temperatures, pressures, and microgravity.^1^ As humans and other mammals have evolved with Earth’s natural gravity, exposure to microgravity with spaceflight can lead to many maladaptive changes, one in particular being a decline in musculoskeletal health and function. The absence of gravity unloads muscles and bones, leading to a reduction in size and strength - akin to aging on Earth. As a result, Space Agencies across the world have focused their attention on developing mitigation strategies to protect musculoskeletal health during space travel, not only to preserve astronaut health and the mission at hand, but also in hopes of developing novel and innovative strategies that may counter aging on Earth.

GSK3 is a ubiquitously expressed and evolutionarily conserved serine/threonine (Ser/Thr) kinase that has two known isoforms: GSK3α and GSK3β, with the latter being dominant in most tissues. It is a constitutively active enzyme, indicating that it has high protein kinase activity under resting conditions, though it can be inhibited through Ser phosphorylation (Ser21, GSK3α; and Ser9, GSK3β).^2^ As both isoforms are structurally similar (95% sequence identity in their catalytic domains^3^), it is not surprising that they also share some functional redundancy, though unique and tissue-specific roles have also been demonstrated.^4^^,^^5^^,^^6^^,^^7^^,^^8^ In general, GSK3 is thought to act as a “brake” in many anabolic pathways, including the Wnt/β-catenin and insulin signaling pathways.^2^ In the context of the musculoskeletal system, GSK3 is a well-known negative regulator of both muscle^9^^,^^10^^,^^11^^,^^12^^,^^13^ and bone mass^2^^,^^10^^,^^14^^,^^15^ that not only slows the anabolic pathways but in many cases can also accelerate the catabolic ones.

With its involvement in the regulation of both bone and muscle mass, it is possible that targeting GSK3 could represent a viable mitigation strategy against the musculoskeletal declines seen with spaceflight. Several lines of evidence found here on Earth are in support of this. We have shown that inhibiting GSK3 with lithium can increase myoblast fusion and differentiation^10^ and promote osteogenic signaling in bones from mice.^10^ Furthermore, patients being treated with lithium for bipolar disorder have been previously shown to have significantly reduced risk of fracture.^16^ In addition, an impressive body of work from Dr. Boris Shenkman’s laboratory has shown that GSK3 is more active in muscles after hindlimb suspension (HLS) – a well-accepted simulated microgravity model – and that its inhibition can attenuate the consequential muscle remodeling (for review readers are referred to ref. 17). Although the evidence from ground-based models suggests that GSK3 may be a viable target against the musculoskeletal decline observed with spaceflight, to our knowledge, there are no studies to date that have investigated this possibility in space. Moreover, the effects of spaceflight, if any, on muscle and bone GSK3 content/signaling remains unknown. This could be an initial first step in firmly establishing GSK3 inhibition as a potential countermeasure.

The purpose of this study was to investigate the effects of spaceflight on murine muscle and bone GSK3 content and activation status. We obtained samples from various missions/payloads where male or female mice were exposed to microgravity for 30–37 days aboard the International Space Station (ISS) or the BION-M1 biosatellite. In addition to this, we characterized the effects of dual muscle-specific GSK3α/β knockdown on muscle size and strength as well as region-specific (tibia, femur, lumbar spine) bone mineral density (BMD) in response to simulated microgravity via HLS. In doing so, we provide evidence in support of targeting GSK3 to enhance bone and muscle health during spaceflight.

## Results

### GSK3β content is reduced in muscles from spacefaring mice

Soleus, extensor digitorum longus (EDL) and tibialis anterior (TA) muscle samples from the flight (F), ground control (GC), and vivarium control (VIV) groups were obtained from the BION-M1 mission as well as the rodent research 1 (RR1), RR9, and RR18 (BuOE and saline-treated) missions through the NASA Life Sciences Data Archive Institutional Scientific Collection Biospecimen Sharing Program. Absolute muscle mass data for the RR9 (soleus and TA) and RR1 (soleus) missions were reported in a recent study published by our group showing significant muscle atrophy.^18^ Muscle mass data for the BION-M1 (soleus and EDL) mission were also reported previously where although both soleus and EDL muscles appeared to be smaller in the F group, there were no significant differences when compared with GC or VIV.^19^ Muscle mass data for RR18 are reported here in Table S1.

**Figure 1:**
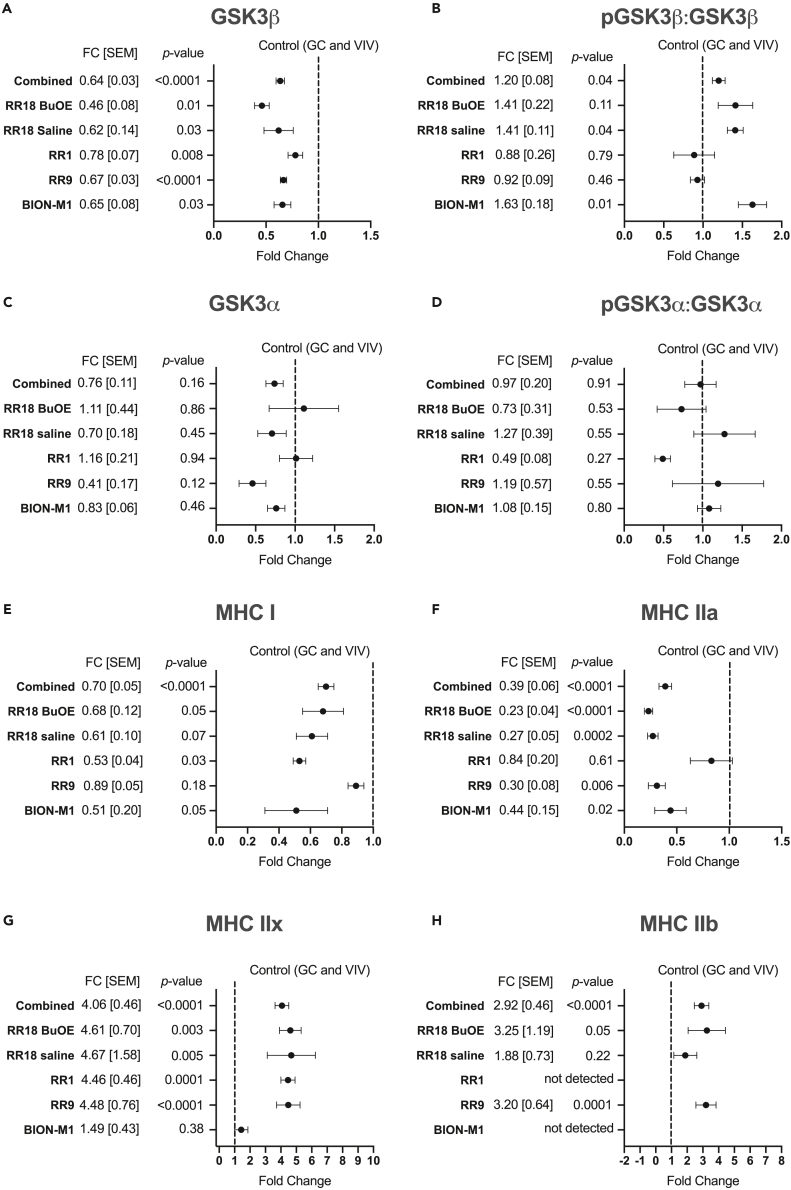
The combined effect of spaceflight across all missions on GSK3 content, phosphorylation status and MHC isoform content in soleus muscles (A) Total GSK3β content. (B) Phosphorylated GSK3β (Ser9) relative to total GSK3β content. (C) Total GSK3α content. (D) Phosphorylated GSK3α (Ser21) relative to total GSK3β content. (E) MHC I content. (F) MHC IIa content. (G) MHC IIx content. (H) MHC IIb content. Fold-change (FC) data were calculated for each mission by dividing the Flight group by the average of the combined GC and VIV groups. The combined dataset represents the mean ± SEM and p value (Student’s *t* test) for all FC data (RR1, RR9, RR18, and BION-M1).

We first characterized the effects of spaceflight on GSK3 content and activation, and although there was no effect of spaceflight in the EDL or TA, we found significant differences in the soleus - a postural muscle that is most affected by microgravity exposure (Figures S1–S7). To summarize the soleus data, we examined the fold-change response in the F group relative to the combined average of the GC and VIV control groups, as no differences were detected between the GC and VIV groups (Figure 1). Across all missions, we observed significant reductions in total GSK3β content (-22-54%) compared with GCVIV control with a combined reduction of 36% (Figure 1A). When examining the overall effect of spaceflight on Ser9 phosphorylated GSK3β content, we found a combined 1.2-fold increase in phosphorylation, however, this was only statistically significant for the BION-M1 and RR18 saline cohorts. Unlike GSK3β content, we did not find a consistent reduction in GSK3α across all missions (Figure 1C). Similarly, we found no combined effect of spaceflight on Ser21 phosphorylation of GSK3α (Figure 1D). Altogether, these findings suggest that spaceflight more prominently affects the GSK3β isoform by reducing its protein levels and, though not consistent for all missions, by also increasing its Ser9 phosphorylation.

**Figure 2:**
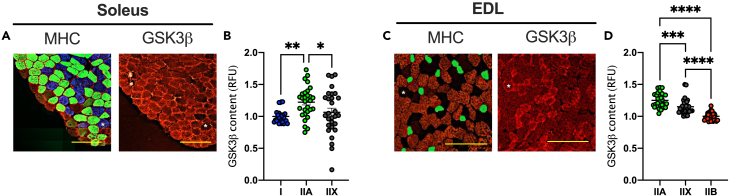
Serial cryosections showing type IIA fiber enrichment of GSK3β (A) Serial 10 μm sections for MHC isoforms (I, blue; IIa, green; IIx, unstained black; and IIb, red) and GSK3β in the soleus. (B) Quantitative comparison of GSK3β across type I, IIA, and IIX fibers in the soleus. Data are expressed relative to type I fibers. (C) Serial 10 μm sections for MHC isoforms (IIa, green; IIx, unstained black; and IIb, red) and GSK3β in the EDL. (D) Quantitative comparison of GSK3β across type IIA, IIX, and IIB fibers in the EDL. Data are expressed relative to type IIB fibers. ∗p < 0.05, ∗∗p < 0.01, ∗∗∗p < 0.001, ∗∗∗∗p < 0.0001, using a one-way ANOVA and a Tukey’s post-hoc test (n = 30 fibers analyzed per group from 3 separate mice). Scale bars are set to 200 μm.

To help explain why GSK3β was lower in soleus muscles after spaceflight, we followed up on our previous findings showing that the fast-twitch EDL muscle has lower GSK3β content compared with the slow-twitch soleus.^13^ We posited that the reduction in GSK3β found with spaceflight would be largely attributed to the slow-to-fast fiber type shift that occurs within the murine soleus.^19^^,^^20^^,^^21^^,^^22^ Across all missions analyzed, there were signs of a slow-to-fast fiber type shift in the soleus after spaceflight with an average 30% and 61% reduction in myosin heavy chain (MHC) I and IIa, respectively (Figures 1E, 1F, S8, and S9). Conversely, we found an average 4-fold and 2.9-fold increase in MHC IIx and IIb isoforms, respectively, in the soleus after spaceflight (Figures 1G, 1H, S8, and S9). The reduction in type IIa is of particular interest as our immunofluorescent microscopy experiments indicate that in the soleus and EDL muscles from otherwise healthy mice (e.g., mobile control), GSK3β is most abundant in the type IIA fibers that are identified with MHC IIa (Figure 2). Thus, the reduction in type IIA fibers found with spaceflight likely accounts for the reduction in GSK3β protein observed with spaceflight.

### Muscle-specific Gsk3 knockdown increases soleus muscle size and strength under simulated microgravity conditions

The reduction in GSK3β observed after spaceflight in the murine soleus could suggest that GSK3 has only a minimal role in the ensuing muscle atrophy and weakness. However, given that our analyses of GSK3 content was conducted in muscle samples obtained after at least 30 days of spaceflight, we next wanted to determine the effects of GSK3 inhibition before microgravity-induced muscle unloading, and more importantly, before any change in fiber type distribution that would result in lowered GSK3β content. To this end, we employed the NASA developed HLS model to simulate microgravity exposure on Earth. To inhibit GSK3, we used a muscle-specific *Gsk3* knockdown (GSK3^mKD^) mouse model previously generated in our lab.^23^ These mice display partial knockdown (-40-50%) of both GSK3 isoforms in the soleus (Figure S10). In turn, these mice have significantly larger soleus muscles - both in absolute and relative to body mass terms (Figure S10). Furthermore, dual X-ray absorptiometry (DXA) scans show that these mice have lowered body fat % and increased lean mass % (Figure S10).

**Figure 3:**
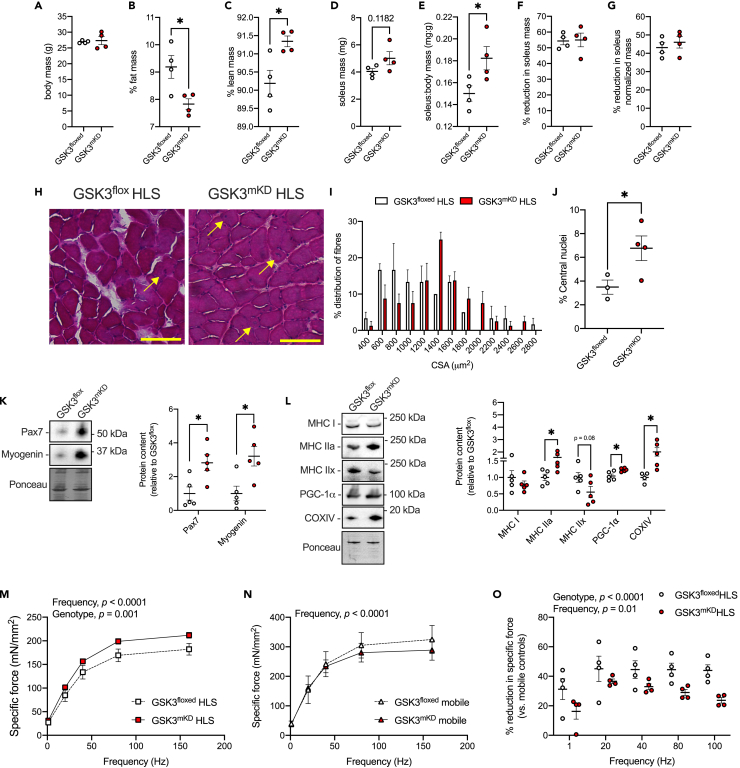
Partial muscle-specific *Gsk3* knockdown (GSK3^mKD^) increases soleus muscle mass, myogenic signaling, and the oxidative phenotype while preserving muscle strength after 7 days of hindlimb suspension (HLS) (A–C) DXA scan analyses showing that GSK3^mKD^ mice have no change in body mass but have lowered % fat mass and increased % lean mass even after 7 days of HLS. (D and E) Absolute and relative (to body mass) soleus muscle weights. (F and G) Percent reduction of absolute and relative soleus muscle weights in GSK3^mKD^ and GSK3^flox^ mice when compared to their respective mobile controls (see Figure S10). (H–J) H&E staining in the soleus shows that GSK3^mKD^ mice have an increased distribution of larger fibers versus GSK3^flox^ mice (rightward shift) and increased centrally located nuclei (see yellow arrows). Scale bars are set to 200 μm; CSA, cross-sectional area. (K) Western blot analysis of myogenic markers Pax7 and myogenin. (L) Western blot analysis of oxidative phenotype markers, MHC I, MHC IIa, PGC-1α, and COXIV as well as the glycolytic MHC IIx. (M) Specific force-frequency curves in soleus muscles from GSK3^mKD^ and GSK3^flox^ control mice after 7 days of HLS. (N) Specific force-frequency curves in soleus muscles from mobile GSK3^mKD^ and GSK3^flox^ control mice. (O) Calculated percent reduction in specific force across stimulation frequencies from GSK3^mKD^ and GSK3^flox^ control mice after 7 days of HLS (compared to their respective mobile controls). For (B, C, E, J, K, L), ∗p < 0.05 using a Student’s *t* test. For (N-O), a two-way ANOVA was used to test the main effects of genotype and frequency. Data are presented as means ± SEM.

After 7 days of HLS, GSK3^mKD^ mice still had lowered % fat mass, elevated % lean mass, and increased soleus:body mass ratio when compared with GSK3^flox^ mice (Figures 3A–3E). However, although GSK3^mKD^ mice had larger soleus muscles after HLS (Figure 3), normalizing the data to mobile controls demonstrated that muscle-specific *Gsk3* knockdown did not attenuate the level of muscle atrophy observed with HLS as both GSK3^mKD^ and GSK3^flox^ mice experienced a ∼50–60% reduction in absolute soleus mass and a ∼40–50% reduction in normalized soleus mass (Figures 3F and 3G). Nonetheless, soleus muscles from GSK3^mKD^ HLS mice were still larger when compared to GSK3^flox^ HLS mice, which was further supported by H&E analysis and a rightward shift in fiber cross-sectional area (CSA) distribution (Figures 3H and 3I). Upon closer inspection, we also found that the GSK3^mKD^ HLS mice had a greater proportion of fibers with centrally located nuclei (Figure 3J), which is indicative of enhanced myogenic repair. Corroborating these findings, myogenic markers Pax7 and myogenin were significantly greater in HLS soleus muscles from GSK3^mKD^ mice versus GSK3^flox^ mice (Figure 3K). Furthermore, we found that soleus muscles from GSK3^mKD^ HLS mice also had greater MHC IIa, peroxisome proliferator-activated receptor-gamma coactivator 1 alpha (PGC-1α), and cytochrome *c* oxidase subunit IV (COXIV) protein levels versus GSK3^mKD^ HLS mice (Figure 3L). With a significant reduction in MHC IIx (Figure 3L), these results provide evidence for an oxidative fiber type shift with muscle-specific *Gsk3* knockdown, which is consistent with our previous findings in mice treated with various pharmacological GSK3 inhibitors.^24^^,^^25^ In terms of soleus muscle strength, we found that after HLS, GSK3^mKD^ mice displayed greater force production across submaximal and maximal frequencies (Figure 3M). This effect was not found in a mobile state (Figure 3N), and therefore, calculating the percent reduction in specific force (HLS mice relative to their respective mobile controls), we found that soleus muscles from GSK3^mKD^ mice had significantly greater preservation (e.g. lower % reduction) of force across submaximal and maximal stimulation frequencies (Figure 3O). Altogether, these results show that targeted GSK3 inhibition before muscle unloading can increase soleus muscle size, myogenic signaling, and the oxidative fiber type while also preserving muscle strength.

### Characterizing GSK3 activation in bones after spaceflight and the effects of muscle-specific GSK3 knockdown on femur, tibia, and lumbar bone mineral density

**Figure 4:**
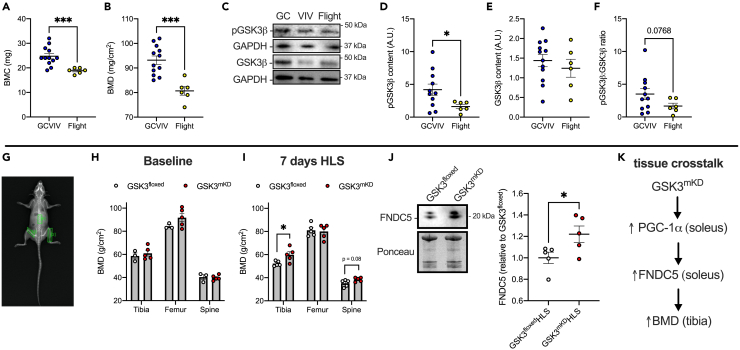
GSK3β phosphorylation and content in femur samples obtained from male RR9 mice (A and B) Bone mineral content (BMC) and bone mineral density (BMD) of the individual bones obtained from a small animal DXA scanner. (C) Representative western blot images of phosphorylated (Ser9) and total GSK3β. (D and E) Western blot analysis of phosphorylated (Ser9) and total GSK3β content normalized to ponceau. (F) GSK3 activation status measured as the ratio of phosphorylated (Ser9) GSK3β relative to total GSK3β. (G) Representative DXA scan showing region-specific analysis of the femur, tibia, and lumbar spine in mice. (H) Region-specific BMD analysis in mobile GSK3^mKD^ and GSK3^flox^ mice measured at baseline. (I) Region-specific BMD analysis in mobile GSK3^mKD^ and GSK3^flox^ mice measured after 7 days of HLS. (J) Western blot analysis of soleus muscle FNDC5 from GSK3^mKD^ and GSK3^flox^ mice measured after 7 days of HLS. (K) Proposed tissue crosstalk between muscle and bone with muscle-specific *Gsk3* deletion leading to an increase in FNDC5 and tibia BMD. ∗p < 0.05, ∗∗∗p < 0.001 using a Student’s *t* test (n = 6–12 per group for (A–F); n = 3–5 per group for (H–J). For (A–F), GC and VIV controls were combined to increase statistical power. All values are presented as means ± SEM.

We then investigated the effects of microgravity exposure on GSK3β content and phosphorylation in femur bones obtained from the RR9 mission. As expected, DXA scan analysis of the femurs showed significantly reduced bone mineral content (BMC) and bone mineral density (BMD) in the F group compared with the combined GCVIV control (Figures 4A and 4B). Additional micro-computed tomography (μCT) analysis of the tibia from these same mice demonstrated decrements in bone structure were apparent in the cortical bone outcomes, whereas no differences in trabecular bone outcomes were evident (Table S2). Specifically, flight mice presented lower cortical area fraction and periosteal perimeter, which was aligned with lower cortical thickness, compared to GCVIV control mice. Western blot analysis revealed a significant reduction in the pGSK3β in the femur compared with GCVIV with no changes in total GSK3β content (Figures 4C–4E). This in turn led to a reduction in the phosphorylation status of GSK3β, and though it did not reach statistical significance (Figure 4F), it is perhaps indicative of greater GSK3β activation in murine bones after spaceflight.

Using the DXA scans from the GSK3^mKD^ and GSK3^flox^ mobile and HLS mice, we then performed bone-specific regional BMD analysis on the tibia, femur, and lumbar spine to determine whether muscle-specific *Gsk3* knockdown could provide positive effects on BMD crosstalk (Figure 4H). In the mobile state, we did not find any differences in region-specific BMD between GSK3^mKD^ and GSK3^flox^ mice (Figures 4G and 4H). However, after 7 days of HLS, we found that tibia BMD was greater in GSK3^mKD^ HLS mice versus GSK3^flox^ HLS mice (Figure 4I). A similar result was found in the lumbar spine; albeit, this was not statistically significant (Figure 4I). In terms of tissue crosstalk, we examined the effects of muscle-specific *Gsk3* knockdown on soleus muscle fibronectin type III domain-containing protein (FNDC5), which is the precursor for the myokine irisin that gets secreted out into circulation with exercise and activation of PGC-1α in muscle.^26^ Irisin produced and secreted from muscle can promote bone strength and mass in mice; and has been shown to limit bone loss associated with HLS.^27^^,^^28^ Here, we demonstrate that the increase in region-specific BMD found with GSK3^mKD^ was associated with a significant increase in FNDC5 in the soleus (Figure 4J). Though our study is limited in that we did not collect serum to measure irisin levels, we think it is possible that muscle-specific *Gsk3* knockdown could positively impact BMD (particularly in the tibia and lumbar spine) by increasing PGC-1α and FNDC5 in muscle (Figure 4K).

## Discussion

Here, we questioned whether GSK3 would be a viable therapeutic target that when inhibited could prevent or limit the muscle and bone loss associated with spaceflight. To this end, we first examined the effects of at least 30 days of spaceflight on GSK3 content and activation status in murine muscle and bone samples. We then supplemented this work with Earth-based HLS experiments in GSK3^mKD^ mice where we assessed muscle size and strength with region-specific BMD.

In the postural soleus muscle, we found that GSK3β but not GSK3α was consistently and significantly reduced in the F group compared with GCVIV, which is in line with previous proteomics analysis from the BION-M1 mission (see Tables S1 and S4 from ref. 19) and indicates that the two isoforms may be differentially regulated during spaceflight. Indeed, these GSK3 isoforms are encoded by separate genes located on two different chromosomes (*Gsk3α,* mouse chromosome 7; and *Gsk3β,* mouse chromosome 18). Furthermore, inhibitory Ser phosphorylation was only enhanced with the GSK3β isoform after spaceflight; albeit not consistently across all missions, whereas Ser phosphorylation of GSK3α was virtually unaltered, which again indicates the two GSK3 isoforms are differentially regulated with spaceflight.

Although the observed reduction in GSK3β content and its increase in inhibitory Ser9 phosphorylation in the soleus may argue against its involvement in the ensuing muscle atrophy and weakness found with muscle unloading and spaceflight, it may just reflect the slow-to-fast fiber type shift, namely a reduction in type IIA fibers which we have shown are particularly enriched with GSK3β. This is consistent with our previous findings showing that GSK3β is highly expressed in the soleus versus the EDL^29^ as the soleus comprises nearly 50% IIA fibers versus the EDL, which only comprises 10% type IIA fibers.^30^ Moreover, we previously found greater Ser9 phosphorylation of GSK3β in the EDL versus the soleus.^29^ Alternatively, or perhaps congruently, it is also possible that these changes in GSK3β content and activation may point toward an adaptive/compensatory response aimed at limiting GSK3 activation to limit muscle atrophy and weakness. Providing additional GSK3 inhibition, preferably before the onset of muscle unloading and the ensuing fiber type changes may then preserve muscle health and function during spaceflight.

To establish whether GSK3 inhibition could potentially preserve muscle size and strength in response to microgravity exposure, we utilized the simulated microgravity HLS model. Our results show that muscle-specific genetic reduction before muscle unloading and the ensuing fiber type shift increased soleus muscle size, myogenic signaling, and preserved muscle strength in the face of HLS. We elected to partially knockdown (-40-50%) both GSK3 isoforms despite the fact that only GSK3β was affected by spaceflight to limit any potential compensatory activation of GSK3α. In a previous study by Pansters et al., sole genetic deletion of *Gsk3β* appeared to have upregulated protein levels of GSK3α in the murine soleus.^31^ In this study, the authors found that genetic knockdown of *Gsk3β* did not attenuate soleus muscle atrophy in response to HLS, however, they did find an acceleration in muscle recovery with reloading.^31^ These findings are consistent with our present results showing that although muscle-specific *Gsk3* knockdown increased soleus muscle size, it did not attenuate the level of muscle atrophy observed with HLS, but it did enhance myogenic signaling. Moreover, our findings extend those from Pansters et al. because they did not examine soleus muscle contractile force. In this respect, GSK3^mKD^ mice displayed greater force production versus GSK3^flox^ mice but only after 7 days of HLS and not in the mobile state. This suggests that the effect of GSK3^mKD^ on muscle strength only manifests after an added stress such as HLS; and it further suggests that in the face of HLS, GSK3^mKD^ mice exhibit a preservation of muscle force production that could be important for spaceflight.

The increase in myogenic signaling observed with GSK3^mKD^ mice is also highly relevant to spaceflight. Though HLS is considered to be the Earth-based analog of spaceflight, the mechanisms behind the ensuing muscle atrophy and weakness with HLS versus actual spaceflight differ in many respects and there is actually very little overlap.^32^ However, one common pathway that is negatively affected in both HLS and spaceflight is myogenesis.^32^ Thus, the observed increase in myogenic signaling in GSK3^mKD^ HLS mice could potentially be translated to spaceflight; however, future studies that investigate the effects of spaceflight directly on these mice are required if not warranted.

The GSK3^mKD^ HLS mice also displayed a promotion toward the oxidative phenotype with a significant increase in MHC IIa, PGC-1α, and COXIV. This suggests that the inhibition of GSK3 not only attenuates muscle atrophy and weakness but may also attenuate the slow-to-fast fiber type shift that could lead to decrements in muscle performance and fatigue resistance. These results are not entirely surprising given that we and others have shown that GSK3 inhibition in muscle can promote the oxidative fiber type and PGC-1α content.^24^^,^^25^^,^^33^^,^^34^^,^^35^^,^^36^ Moreover, the increase in PGC-1α content in muscle can provide benefits outside of muscle by promoting the expression, production, and release of FNDC5 and irisin. Irisin is a myokine secreted from muscle that can positively impact bone mass and strength even in the face of HLS.^27^^,^^28^^,^^37^ Specifically, genetic deletion of irisin in mice severely compromises bone strength and mass with an increased number of osteoclasts,^27^ whereas, irisin treatment prevented bone loss with HLS and accelerated bone recovery in response to reloading in mice.^28^ Here, we show that muscle-specific *Gsk3* knockdown increased FNDC5 in muscle while also increasing BMD in the tibia and in the lumbar spine (though not statistically significant in the latter, p = 0.08). Combined with our results showing that GSK3β is overactive in bones (e.g., less inhibitory Ser phosphorylation) from obtained mice subjected to spaceflight, our study provides ample evidence in support of inhibiting GSK3 for the promotion of musculoskeletal health during spaceflight.

In human astronauts, the inhibition of GSK3 before and during spaceflight could be achieved with pharmacological inhibitors such as lithium (Li), which is an already FDA-approved drug. It is most commonly used for the treatment of bipolar disorder; however, because Li also exerts adverse effects when taken at relatively high doses (serum [Li] concentration exceeding 1.0 mM) for a prolonged period of time, it must be prescribed within a narrow therapeutic range (0.5–1.0 mM serum concentration) (for review see ref. 38). Conversely, we have shown that treating mice with lower doses of Li (serum [Li] of 0.02 mM) increases soleus and EDL muscle specific force production while also promoting PGC-1α and the oxidative fiber type.^25^ In cell culture, we have also shown that low dose Li supplementation augments myogenic differentiation and fusion,^29^ which could have implications for spaceflight. In fact, pre-treating mice with a low dose of Li (10 mg/kg/day via drinking water) for 4 weeks before unloading the soleus muscle via tenotomy, inhibited GSK3 and increased soleus muscle size even in the face of tenotomy surgery (Figure S10). In addition to muscle, Li has been shown to have osteoprotective effects.^2^^,^^10^^,^^14^^,^^15^^,^^16^ In a recent and large epidemiological study, it was found that patients living with bipolar disorder and receiving Li treatment had reduced risk of developing osteoporosis.^39^ This is consistent with our findings in mice whereby low dose Li supplementation augmented osteogenic signaling by inhibiting GSK3, which in turn activated the anabolic Wnt/β-catenin pathway.^10^ It should be noted that Li has several osteoprotective effects acting on other pathways such as the phosphatidylinositol 3-kinase (PI3K)/protein kinase B (Akt) and bone morphogenetic protein-2 (BMP-2) transduction pathways (for review see^40^). Altogether, the results from our study warrant future investigations with spaceflight and/or HLS to examine the effects of pharmacological inhibition of GSK3 before and during microgravity exposure to further establish whether targeting GSK3 can exert musculoskeletal protection against spaceflight. Undoubtedly, other GSK3 inhibitors aside from Li that are more specific to GSK3 would be important to test and could include drugs like tideglusib, which is the most clinically advanced GSK3 inhibitor currently being tested for other diseases and conditions.^41^^,^^42^ We have recently shown that tideglusib treatment can preserve muscle size, strength, and quality in dystrophic mice.^24^

The limitations of our study include low sample size, particularly with the RR1 and BION-M1 missions. Although the RR1 mission was conducted in female mice, the limitation in sample size prevented us from truly examining any potential influence of biological sex. Future studies, with age-matched male and female mice, preferably part of the same mission, are required to better elucidate the potential sex-specific effects of spaceflight on the musculoskeletal system. Our study was also limited in that we were not able to obtain bone samples from all missions, and thus our analysis was restricted to the RR9 mission. Within this mission, μCT analyses of the tibia indicated that cortical bone was primarily affected by spaceflight. However, we suspect that limitations in sample size prevented us from detecting a statistically significant effect on trabecular bone. Indeed, previous μCT analyses in the lumbar vertebrae and femur from the BION-M1 mission showed significant reductions in trabecular bone outcomes in the F group versus GC and VIV controls.^43^ In muscle, where we obtained samples from RR9, RR1, RR18, and BION-M1 missions, we utilized a “meta-analytical” approach with the fold-change response as the common measure of effect size. However, differences in experimental conditions (i.e., food, temperature, pressure, strain differences, age differences, and etc.) should not be discounted. These differences could have contributed to the differences in phosphorylation status of GSK3, where we found significant increases in Ser phosphorylation of GSK3 in BION-M1 and RR18 F soleus muscles but not in any other soleus muscles from RR1 or RR9. Finally, our study is limited in that we did not assess the effects of bone-specific *Gsk3* deletion on BMD and strength in response to HLS. Future studies should generate these mice and perform these experiments to firmly establish whether GSK3 inhibition before simulated microgravity exposure can protect against bone loss. Nevertheless, our findings demonstrate that inhibiting GSK3 specifically in muscle may exert positive effects on the bone thereby highlighting a muscle-to-bone connection that would likely be activated with whole-body GSK3 inhibition afforded through pharmacological means.

In summary, our study shows that GSK3 content and activation status are altered in murine soleus and bone samples obtained after at least 30 days of spaceflight. Although GSK3β content was reduced after spaceflight, our results with the simulated microgravity HLS model shows that inhibiting GSK3 before muscle unloading via muscle-specific *Gsk3* deletion can increase muscle mass, myogenic signaling, and can preserve muscle force production. Moreover, muscle-specific *Gsk3* deletion may also help to maintain bone mass by increasing PGC-1α and FNDC5; and thus, future studies aboard the ISS are warranted to examine whether these mice are protected against spaceflight-induced declines in musculoskeletal health. In addition, future studies that examine the effects of pharmacological, and thus, whole-body GSK3 inhibition before and during spaceflight should also be done in mice. Collectively, our present study represents an important first step in hopes of enabling longer duration spaceflight missions to Mars – possibly through GSK3 inhibition.

## STAR★Methods

### Key resources table

**Table:** 

REAGENT or RESOURCE	SOURCE	IDENTIFIER
**Antibodies**		

GSK3β	Cell signaling	Cat# 9315; RRID: AB_490890
GSK3β (Ser9 phosphorylated)	Cell signaling	Cat# 9322; RRID: AB_2115196
GSK3α	Cell signaling	Cat# 4818; RRID: AB_10831511
GSK3α (Ser9 phosphorylated)	Cell signaling	Cat# 9316; RRID: AB_659836
PGC-1α	Millipore	Cat# ST1204; RRID: AB_10807905
COXIV	Abcam	Cat# ab16056; RRID: AB_443304
FNDC5	Abcam	Cat# ab131390
MHCI	DHSB	Cat# BA-F8; RRID: AB_10572253
MHC IIa	DHSB	Cat# SC-71; RRID: AB_2147165
MHC IIx	DHSB	Cat# 6H1; RRID: AB_1157897
MHC IIb	DHSB	Cat# BF-F3; RRID: AB_2266724
Pax7	DHSB	Cat# APAX7; RRID: AB_2299243
Myogenin	DHSB	Cat# Myog; RRID: AB_2722260
Anti-mouse HRP	Cell Signaling	Cat# 7076; RRID: AB_330924
Anti-rabbit HRP	Cell Signaling	Cat# 7074; RRID: AB_2099233
Alexa Fluor 647 anti-rabbit	ThermoFisher	Cat# A27040; RRID: AB_2536101
Alexa Fluor 350 anti-mouse	ThermoFisher	Cat# A-21140; RRID: AB_2535777
Alexa Fluor 488 anti-mouse	ThermoFisher	Cat# A-21121; RRID: AB_2535764
Alexa Fluor 555 anti-mouse	ThermoFisher	Cat# A-21426; RRID: AB_2535847

**Biological samples**		

RR9 soleus and tibialis anterior	NASA Biospecimens Sharing Program	N/A
RR1 soleus	NASA Biospecimens Sharing Program	N/A
RR18 soleus	NASA Biospecimens Sharing Program	N/A
BION-M1 soleus and extensor digitorum longus	Dr. Fabrice Bertile (Strasbourg, France)	N/A

**Experimental models: Organisms/strains**		

GSK3α/β floxed mice	Brock University	N/A
ACTA-1 Cre mice	Jackson Laboratories and now housed at Brock University	N/A

**Software and algorithms**		

Molecular Devices SoftMax Pro	Molecular Devices	https://www.moleculardevices.com/products/microplate-readers/acquisition-and-analysis-software/softmax-pro-software?cmp=7010g000000nNCH&utm_source=AdWords&utm_medium=cpc&utm_campaign=MPR-Brand_CANADA&utm_adgroup={adgroup}&utm_location=9000776&utm_keyword=molecular%20devices%20softmax%20pro&utm_device=c&utm_devicemodel=&utm_placement=&utm_adpostion=&utm_target=&utm_network=g&utm_creative=413034758091&gclid=CjwKCAjw0dKXBhBPEiwA2bmObfQYfxhI9dqx2kDoEaENs2bFYuBzCML-NluCxhjnZ3efLgFFif0rvhoCsnYQAvD_BwE
ImageLab	BioRad	https://www.bio-rad.com/fr-ca/product/image-lab-software?ID=KRE6P5E8Z
Graphpad Prism 8	Graphpad Prism	https://www.graphpad.com/scientific-software/prism/
ImageJ	NIH	https://imagej.nih.gov/ij/download.html
NRecon V.1.7.3.1 software	Bruker microCT	N/A
Osteosys software for INSIGHT small animal DXA scanner	Scintica	https://www.scintica.com/wp-content/uploads/2022/03/FAQ-iNSiGHT-DXA_v0122-1.pdf
Aurora Scientific 600A software	Aurora Scientific	https://aurorascientific.com/products/muscle-physiology/muscle-daq-software/600a-real-time-muscle-system/

### Resource availability

#### Lead contact

Further information and requests for resources and reagents should be directed to and will be fulfilled by the lead contact, Val A. Fajardo (vfajardo@brocku.ca).

#### Materials availability

This study did not generate new unique reagents.

### Experimental models and subject details

#### Muscle samples from RR1, RR9, RR18, and BION-M1 missions

Soleus, EDL, and TA muscle samples were obtained from the BION-M1 mission as well as the RR1, RR9, and RR18 missions through the NASA Life Sciences Data Archive Institutional Scientific Collection Biospecimen Sharing Program. For all missions, we were provided with 3 experimental groups: 1) Flight (F) group housed in specialized mission hardware; 2) Ground control (GC) housed in specialized mission hardware here on Earth; and 3) Vivarium (VIV) control housed in standard laboratory cages and conditions. For the BION-M1 mission, male C57BL/6N (19–20 week old at time of launch) mice were maintained in high-orbit (∼550 km) for 30 days in specialized flight modules.^44^ For the NASA missions, male C57BL/6J (RR9 and RR18, 10 weeks of age at time of launch) and female C57BL/6J (RR1, 16 weeks of age at time of launch) mice were acclimated and then launched in a specialized transporter and transferred to Rodent Habitats^45^ on board the International Space Station (ISS). Additional details on housing and tissue collection/preparation can be found elsewhere for RR1, RR9, and BION-M1.^18^^,^^19^ For the RR18 mission, flight mice were aboard the ISS for 30 days with weekly injections of either sterile saline (0.9%) or the antioxidant metalloporphyrin (MnTnBuOE-2-PyP5+, herein referred to as BuOE). The mice used in this study were part of a live animal return cohort where tissue dissection occurred 1 day after landing. Soleus muscles were collected and stored in RNAlater at −80°C, prior to being homogenized in PMSF buffer (250 mM Sucrose, 5 mM HEPES, 0.2 mM PMSF, 0.2% NaN_3_ (pH 7.5)). We did not find any differences in GSK3 content or MHC isoform expression between the two saline and BuOE cohorts, and thus we elected to keep them separate in our analysis.

In addition to the F, GC, and VIV groups, we also received two cohort control groups from the RR9 mission. Owing to Hurricane Irma (September 2017), the original RR9 GC and VIV experiments were terminated early. GC and VIV experiments were replicated in May 2018 using the same strain of mice used for the flight experiment. With the new GC and VIV groups, an additional set of mice were used as cohort controls to assess variation because of differences in time. That is, the mice originally dedicated to serve as the VIV group in 2017 were labeled as cohort 1, and another set of age- and sex-matched mice were run as cohort 2 under similar treatments in 2018. Thus, differences in GSK3 expression and activation were first examined in R9 cohort controls to determine if a normalizing factor would be required. Importantly, we found no differences in GSK3 phosphorylation in the TA muscles from Cohort Control 1 (CC1) and Cohort Control 2 (CC2) (Figure S12). We also found no significant differences between cohort controls (CC1 vs. CC2) in any of the bone outcomes measured (Table S3).

#### Animals

Skeletal muscle-specific *Gsk3* partial knockdown mice of a C57BL/6J background were generated by crossing GSK3α/β floxed mice (kindly donated by Dr. Virginia Lee, University of Pennsylvania) with ACTA-1-Cre mice (Jackson laboratories) under the control of the skeletal muscle α-actin promoter.^23^ Heterozygously floxed GSK3α/β mice also heterozygously expressing ACTA-1 Cre were considered GSK3^mKD^, whereas heterozygously floxed GSK3α/β mice without ACTA-1 Cre were considered the flox control (GSK3^flox^). For the lithium feeding experiments, male 3–6 month old C57BL/6 mice were used. All mice were fed a standard chow diet (2014 Teklad global, 14% protein rodent maintenance diet, Harlan Teklad, Mississauga, ON), and were kept on a 12-h light: 12-h dark cycle with *ad libitum* access to food and water through the entirety of the study. Mice were housed at a temperature of 22–24°C for the duration of the study.

#### Ethics statement

This study utilized murine muscle samples provided to us through the NASA Life Sciences Data Archive Institutional Scientific Collection Biospecimen Sharing Program. All animal procedures performed were approved by the Institutional Animal Care and Use Committees (IACUC) for flight at the NASA Ames Research Center (ARC) and the Kennedy Space Center (KSC), and the methods were carried out in accordance with relevant guidelines and regulations. Experimental protocols for the GSK3^mKD^ and lithium feeding experiments were approved by the Brock University Animal Care Committee (files #17-06-03, #20-07-01, and #21-06-02) and were in compliance with the Canadian Council on Animal Care.

### Method details

#### Hindlimb suspension

For the hindlimb suspension (HLS) experiments, the hindlimbs of male GSK3^mKD^ and GSK3^flox^ (4–6 month old) mice were suspended using a taping method where the tails of mice were taped to a copper ring attached to a steel bar that was mounted on top of a modified mouse cage. For these experiments, mice were pair-housed in the cages and had as libitum access to food and water. After 7-day of HLS, all mice were sacrificed via cervical dislocation (under isoflurane) and their soleus muscles were collected.

#### Lithium feeding and tenotomy surgery

For the lithium feeding study, C57BL/6 male mice (3–6 months of age) were randomly divided into a control or lithium group. The lithium group received a dose of 10 mg/kg/day of LiCl via their drinking water as previously described^10^^,^^23^^,^^25^^,^^46^ whereas the control group received drinking water without LiCl supplementation. The total duration of treatment was 6 weeks. On the fourth week, all mice underwent the tenotomy surgery as previously described.^47^^,^^48^^,^^49^ Briefly, mice were anesthetized with vaporized isofluorane, prior to severing the soleus and gastrocnemius tendons from one leg. A sham surgery was conducted on the contralateral leg to serve as an internal control. Both wounds on both legs were closed with silk suture and mice were returned to their home cages, where LiCl or control treatment was maintained for an additional two weeks prior to euthanization. After euthanizing the mice via cervical dislocation, soleus muscles were collected for future analyses.

#### DXA scanning of individual bones

A small animal DXA scanner (OsteoSys InSIGHT, Scintica) was used to scan the individual femur bones collected from the RR9 mission. Overall coefficient of variation of our analysis was 1.7% for BMD and 3.2% for BMC. In addition, we also performed region-specific BMD analysis for the tibia, femur, and lumbar spine from mobile and HLS GSK3^mKD^ and GSK3^flox^ mice.

#### Soleus muscle contractility

Soleus muscle contractility was assessed as previously described^25^^,^^47^ at 25°C using an Aurora Scientific contractile apparatus with a biphasic simulator (model 305B and 701B). Soleus muscles were subjected to a force frequency curve (1, 20, 40, 80 and 160) with a sampling rate of 1000 Hz.^50^ For force-frequency analysis, peak isometric force was obtained. Cross-sectional area (CSA) was calculated using the following formula: CSA = m/l∗d∗(L_f_/L_o_), where m, muscle mass (mg); l, muscle length (mm); d, mammalian skeletal muscle density (1.06 mg/mm^3^); Lf/Lo is the fiber length-to-muscle length ratio (0.71 for soleus). Specific force was calculated by normalizing peak isometric force by CSA.

#### Western blotting

A bicinchoninic acid (BCA) assay was used to determine sample protein concentration using an M2 Molecular Devices Plate Reader (Molecular Devices). Western blotting was performed to determine GSK3β, phosphorylated (p)-GSK3β, GSK3α, (p)-GSK3α, β catenin, MHC I, MHC IIa, MHC IIx, MHC IIb. Antibodies from pGSK3β (9336), total (t)-GSK3-β (9315), pGSK3α (9316), tGSK3α (4818), β-Catenin (8480) were obtained from Cell Signaling Technology (Danvers, MA, USA). MHC I (BA-F8), IIa (SC-71), IIx (6H1), and IIb (BF-F3) antibodies as well as the antibodies for Pax7 (AB_528428) and myogenin (F5D) were obtained from the Developmental Studies Hybridoma Bank (University of Iowa). The antibodies for FNDC5 and COXIV were obtained from Abcam (ab13190 and abS16056, respectively) and the antibody for PGC-1α was obtained from Millipore (ST1204). All Western blots used BioRad TGX 4–15% gradient gels (4561086: BioRad) and PVDF membranes. Samples were then prepped and equally loaded for gel electrophoresis. Transfer was conducted via BioRad Transblot Turbo. Following transfer, membranes were blocked in 5% milk in TBST for 60 min or BioRad Every Blot blocking buffer (12010020) for 10 min. Following blocking, the appropriate primary antibody was appropriately diluted and added during an overnight incubation at 4°C. After incubation with appropriate primary antibodies membranes were washed 3 times for 5 min with TBST then incubated for 60 min with the analogous secondary anti-mouse (7076; Cell Signaling Technology; MHC I, IIa, IIx, IIb) or anti-rabbit (7074; Cell Signaling Technology; p-GSK3α, GSK3α p-GSK3β, GSK3β, β-catenin) antibody both conjugated to horseradish peroxidase. Upon secondary incubation, membranes were washed another 3 times with TBST for 5-min each. Millipore Immobilon (#WBKLSO500) chemiluminescent substrate was added and then visualized using a BioRad ChemiDoc MP for protein detection. Ponceau staining was used for protein normalization (59803; Cell Signaling Technology).

#### GSK3 fiber type analysis

To assess the fiber type expression of GSK3β, we performed immunofluorescent microscopy experiments on serial 10 μm cryosections obtained from O.C.T. (optimal cutting temperature) embedded soleus and EDL muscles from n = 3 mobile wild-type C57BL/6J male mice. One serial cryosection underwent immunofluorescent fiber type staining as previously described to demarcate the various fiber types with MHC I, IIa, IIx, and IIb isoforms.^30^^,^^47^ The other serial cryosection underwent immunofluorescent staining with a primary antibody for GSK3β (9315, Cell Signaling, 1:2500 dilution) and an anti-rabbit Alexa Fluor 647 fluorescent secondary antibody (A27040, ThermoFisher Scientific, 1:5000 dilution). Once fiber types were identified with MHC isoform expression/fluorescence, GSK3β was quantified by converting the image to grayscale and quantifying the mean gray value with ImageJ. Within a single muscle, 10 of each fiber type were randomly analyzed.

#### Histological analysis

Soleus muscles embedded in O.C.*T. matrix* from GSK3^mKD^ and GSK3^flox^ HLS mice were processed into 10 μm cryosections prior to undergoing H&E staining. For each muscle, 2–3 images were randomly taken and the CSA of 20 fibers per mouse (n = 3–4 per group) were obtained using ImageJ. For central nuclei counts, the total number of fibers from the H&E images were counted, ranging from 150 to 230 fibers per image and the number of fibers with central nuclei calculated as a percent of the total number of fibers within the image.

#### Micro-computed tomography bone imaging

Micro-computed tomography (μCT, SkyScan 1176 V.1.1 build 12, Bruker microCT, Belgium) was performed as previously described.^51^ Following euthanasia, the right tibia was excised, cleaned of surrounding tissue, and wrapped in parafilm wax to prevent moisture loss throughout the duration of the 180-degree high resolution (9 μm) scan. A 0.25 mm aluminum filter was applied. The beam strength was set at 45 kV and 545 μA for an exposure time of 850 ms. Resulting images were reconstructed (smoothing kernel = 2, ring artifact reduction = 8, beam hardening = 30%, and dynamic image range = 0.000–0.155; NRecon V.1.7.3.1 software, Bruker microCT, Belgium) into 3D images and reoriented in anatomical position (DataViewer V.1.5.6.2 64-bit software, Bruker microCT, Belgium) for consistency when selecting the regions of interest. Quantification of trabecular and cortical bone structure outcomes was performed at the proximal tibia and tibia midpoint, respectively.

#### Assessment of trabecular bone structure at the proximal tibia

Trabecular bone region of interest began 0.855 mm from the point in which the primary spongiosa disconnects and formation of the growth plate is evident and expanded 0.675 mm toward the tibia midpoint. Trabecular bone was manually delineated from the cortical bone and quantified using an adaptive thresholding of 49 to determine total volume, bone volume, bone volume fraction, trabecular thickness, trabecular separation, trabecular number, degree of anisotropy, and connectivity density.

#### Assessment of cortical bone structure at the tibia midpoint

For cortical bone structure analysis, the tibia midpoint was used to define the region of interest (0.450 mm toward the proximal and distal ends; total height of 0.900 mm). Cortical bone structure outcomes, including total cross-sectional area, cortical area, cortical area fraction, cortical thickness, periosteal perimeter, endocortical perimeter, medullary area, and eccentricity, were calculated using an adaptive threshold of 67.

### Quantification and statistical analysis

#### Statistical analyses

To examine the combined effect of spaceflight on GSK3 content and phosphorylation, fold-change values were calculated for each mission relative to the average of both GC and VIV controls. The fold-change values were compared with GCVIV controls using a Student’s *t* test. For the western blot data across the individual experiments (Figures S1–S3, S5, and S6), a one-way ANOVA or non-parametric Kruskal Wallis test with a Dunnet’s post-hoc test was used to strictly compare GC vs. Flight and VIV vs. Flight. This method was chosen since no significant differences between GC and VIV for any outcome measure related to muscle were detected for any mission. Similarly, for the bone analysis (DXA, μCT, and western blot analyses) GC and VIV controls were combined as there were no differences between the two groups. This was done to increase statistical power. A Student’s *t* test was then used to make comparisons between F and GCVIV. For the GSK3^mKD^ experiments, most comparisons were made Student’s *t* test unless otherwise stated where a two-way ANOVA was used. For the lithium and tenotomy experiments, a two-way ANOVA was also used to test the main effects of lithium treatment and tenotomy. All data are presented as mean +/− standard error unless stated otherwise. A Shapiro-Wilk test was used to test for normality for both studies. Statistical tests were conducted through GraphPad Prism 8 Software. Any statistical outliers detected using the ROUT test (Q = 5%) on GraphPad Prism were removed prior to analysis. A p ≤ 0.05 was used to determine statistical significance.

## Data Availability

• All data reported in this paper will be shared by the lead contact upon request. • This paper does not report original code. • Any additional information required to reanalyze the data reported in this paper is available from the lead contact upon request.
