# Supplementary material for: Toward countering muscle and bone loss with spaceflight: GSK3 as a potential target
Source: iScience. 2023 Jun 8;26(7):107047. doi: 10.1016/j.isci.2023.107047 (PMC10285634; doi:10.1016/j.isci.2023.107047)
Supplement: Document S1. Figures S1–S12 and Tables S1–S3 [file mmc1.pdf]

iScience, Volume *26*

## Supplemental information

# Toward countering muscle and bone loss with spaceflight: GSK3 as a potential target

**Ryan W. Baranowski, Jessica L. Braun, Briana L. Hockey, Jenalyn L. Yumol, Mia S. Geromella, Colton J.F. Watson, Nigel Kurgan, Holt N. Messner, Kennedy C. Whitley, Adam J. MacNeil, Guillemette Gauquelin-Koch, Fabrice Bertile, William Gittings, Rene Vandenboom, Wendy E. Ward, and Val A. Fajardo**

**Towards countering muscle and bone loss with spaceflight: GSK3 as a potential target**

Ryan W. Baranowski[1,2], Jessica L. Braun[1,2], Briana L. Hockey[1,2], Jenalyn L. Yumol[1,2], Mia S. Geromella[1,2], Colton J.F. Watson[3], Nigel Kurgan[1,2], Holt N. Messner[1,2], Kennedy C. Whitley[1,2,], Adam J. MacNeil[3], Guillemette Gauquelin-Koch[4], Fabrice Bertile[5], William Gittings[1,2], Rene Vandenboom[1,2], Wendy E. Ward[1,2], Val A. Fajardo[1,2]*

1 Department of Kinesiology, Brock University, St. Catharines, ON, Canada.
2 Centre for Bone and Muscle Health, Brock University, St. Catharines, ON, Canada.
3 Department of Health Sciences, Brock University, St. Catharines, ON, Canada
4 French Space Agency, Centre National d'Etudes Spatiales (CNES), Paris, France.
5 Hubert Curien Pluridisciplinary Institute (IPHC), CNRS, Strasbourg University, Strasbourg, France.

**SUPPLEMENTARY MATERIAL**

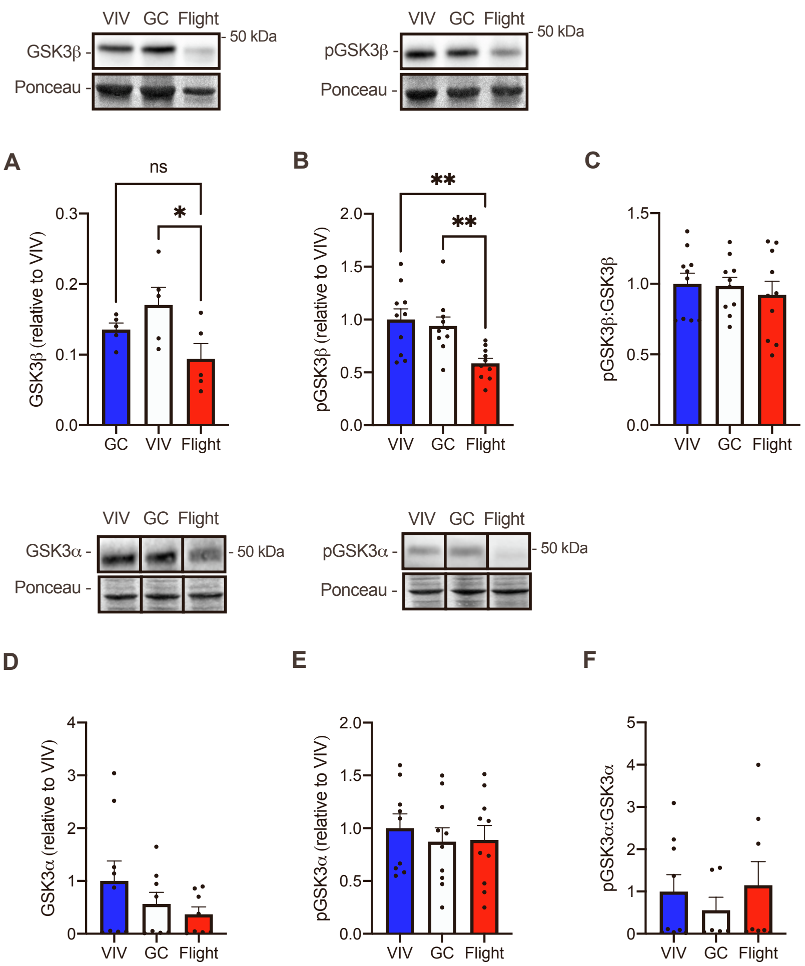


**Supplementary Fig. 1. GSK3 content and phosphorylation status in soleus muscles from GC, VIV and Flight mice from the RR9 soleus, [Related to Figure 1]. A-C)** Total, phosphorylated (Ser9), and the phosphorylated:total GSK3β ratio in Flight and control groups. **D-F)** Total, phosphorylated (Ser21), and the phosphorylated:total GSK3α ratio in Flight and control groups. Data are presented as means ± SEM, $^{**}p<0.01$,$^{****}p<0.0001$, using a one-way ANOVA or Kruskal-Wallis (for D and F) test with Dunnett's post-hoc test (n=8-10 per group). For D and E, the representative blots were loaded in GC, VIV, Flight on the same gel, and displayed here as VIV, GC, and Flight.

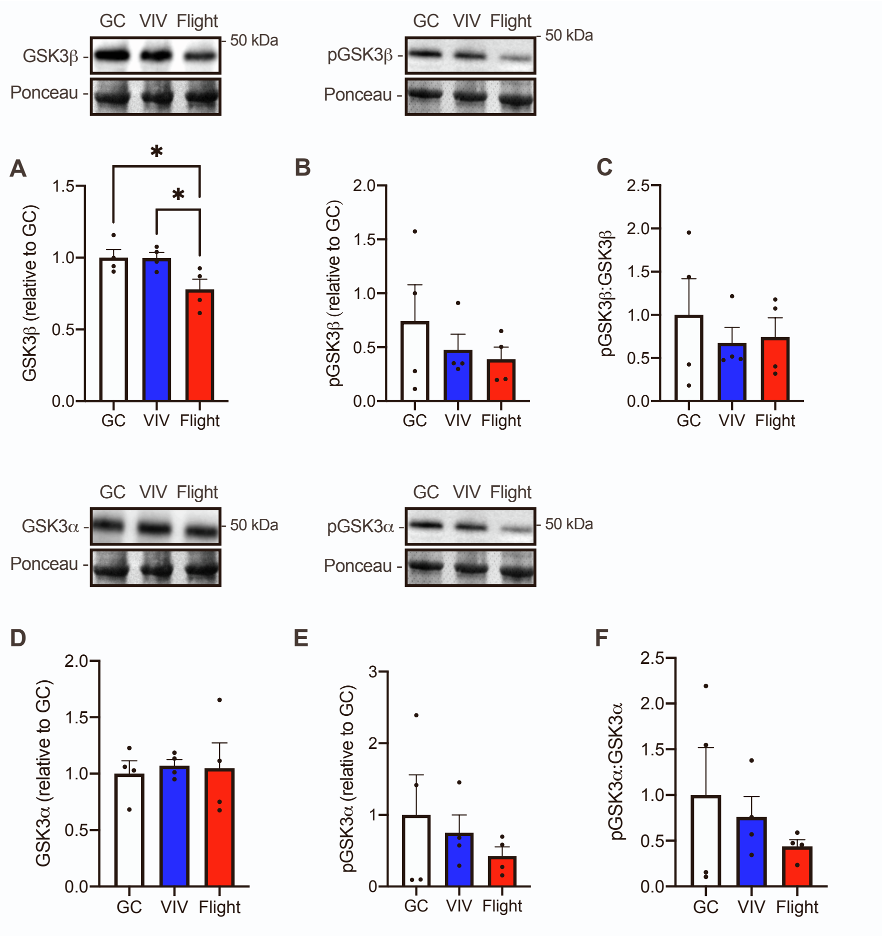


**Supplementary Fig. 2. GSK3 content and phosphorylation status in soleus muscles from GC, VIV and Flight mice from the RR1 mission, [Related to Figure 1]. A-C)** Total, phosphorylated (Ser9), and the phosphorylated:total GSK3β ratio in Flight and control groups. **D-F)** Total, phosphorylated (Ser21), and the phosphorylated:total GSK3α ratio in Flight and control groups. Data are presented as means ± SEM, Data are presented as means ± SEM, *$p<0.05$, using a one-way ANOVA or Kruskal-Wallis (for B and C) test with Dunnett's post-hoc test, n = 4 per group

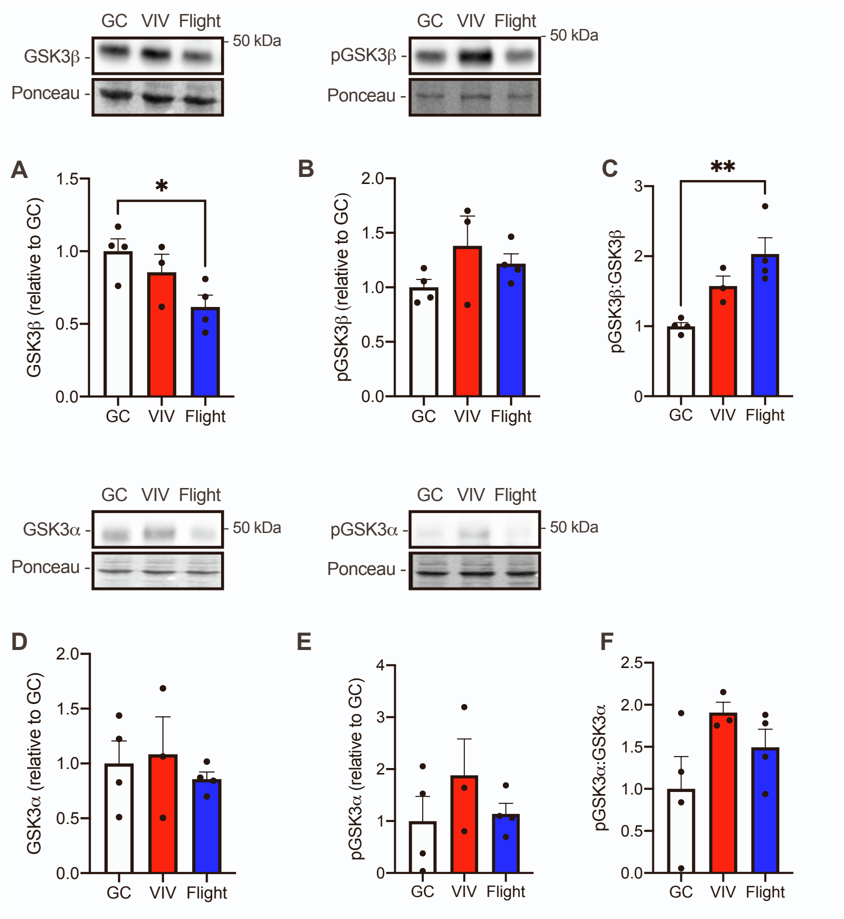


**Supplementary Fig. 3. GSK3 content and phosphorylation status in soleus muscles from GC, VIV and Flight mice from the BION-M1 mission, [Related to Figure 1]. A-C)** Total, phosphorylated (Ser9), and the phosphorylated:total GSK3β ratio in Flight and control groups. **D-F)** Total, phosphorylated (Ser21), and the phosphorylated:total GSK3α ratio in Flight and control groups. Data are presented as means ± SEM, *$p<0.05$, **$p<0.01$, using a one-way ANOVA with Dunnett's post-hoc test, n = 3-4 per group.

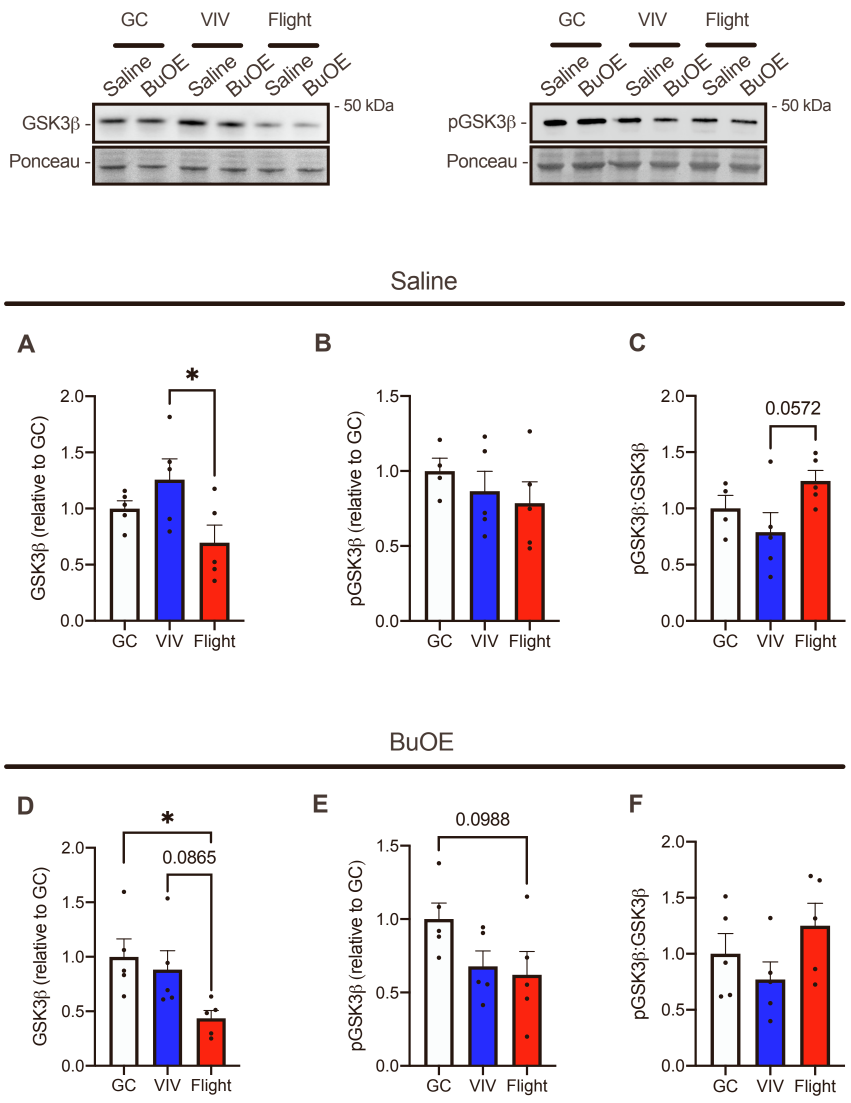


**Supplementary Fig. 4. GSK3β content and phosphorylation status (Ser9) in soleus muscles from GC, VIV and Flight mice from the RR18 mission, [Related to Figure 1]. A-C)** Total, phosphorylated (Ser9), and the phosphorylated:total GSK3β ratio in Flight and control groups from saline-treated mice. **D-F)** Total, phosphorylated (Ser9), and the phosphorylated:total GSK3β ratio in Flight and control groups from BuOE-treated mice. Data are presented as means ± SEM, *$p<0.05$, **$p<0.01$, using a one-way ANOVA with Dunnett's post-hoc test, n = 4-5 per group.

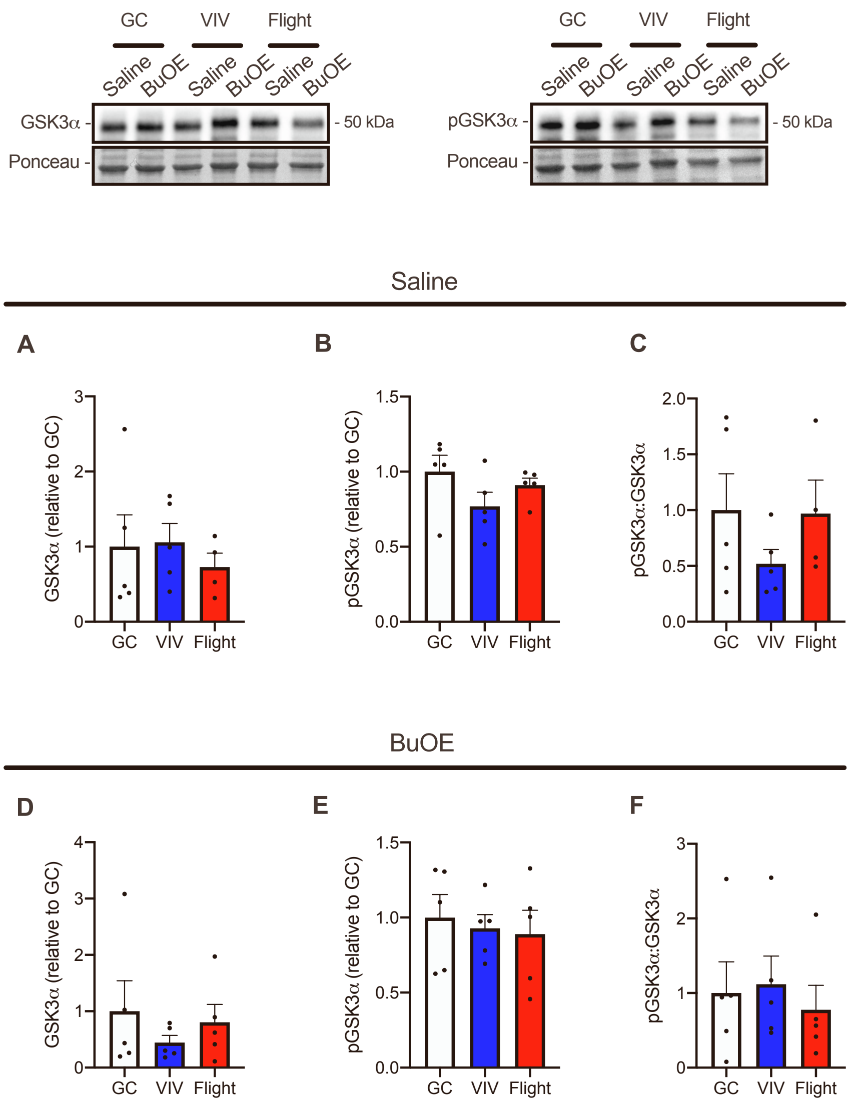


**Supplementary Fig. 5. GSK3α content and phosphorylation status (Ser21) in soleus muscles from GC, VIV and Flight mice from the RR18 mission, [Related to Figure 1]. A-C)** Total, phosphorylated (Ser21), and the phosphorylated:total GSK3α ratio in Flight and control groups from saline-treated mice. **D-F)** Total, phosphorylated (Ser21), and the phosphorylated:total GSK3α ratio in Flight and control groups from BuOE-treated mice. Data are presented as means ± SEM.

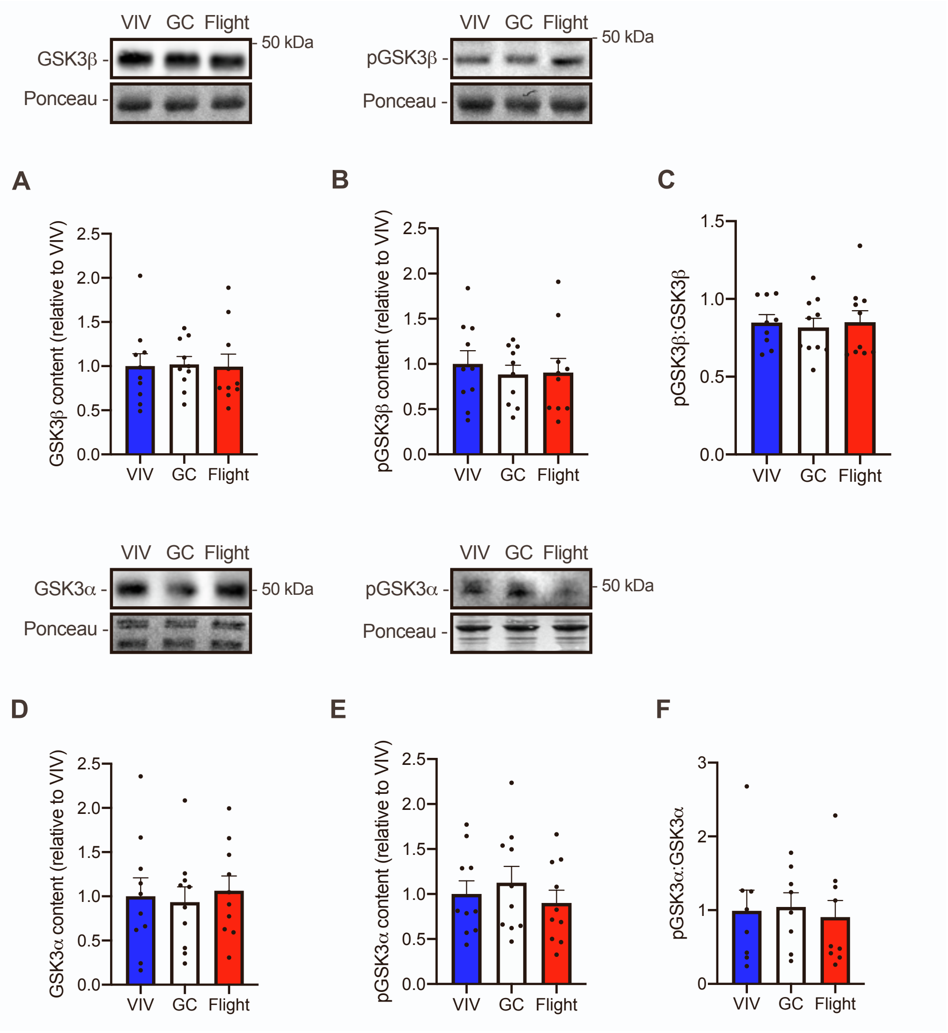


**Supplementary Fig. 6. GSK3 content and phosphorylation status in TA muscles from GC, VIV and Flight mice from the RR9 mission, [Related to Figure 1]. A-C)** Total, phosphorylated, and the phosphorylated:total GSK3β ratio in Flight and control groups. **B)** Phosphorylated GSK3β (Ser9) content. **D-F)** Total, phosphorylated, and the phosphorylated:total GSK3α ratio in Flight and control groups. Data are presented as means ± SEM, n = 8-10 per group.

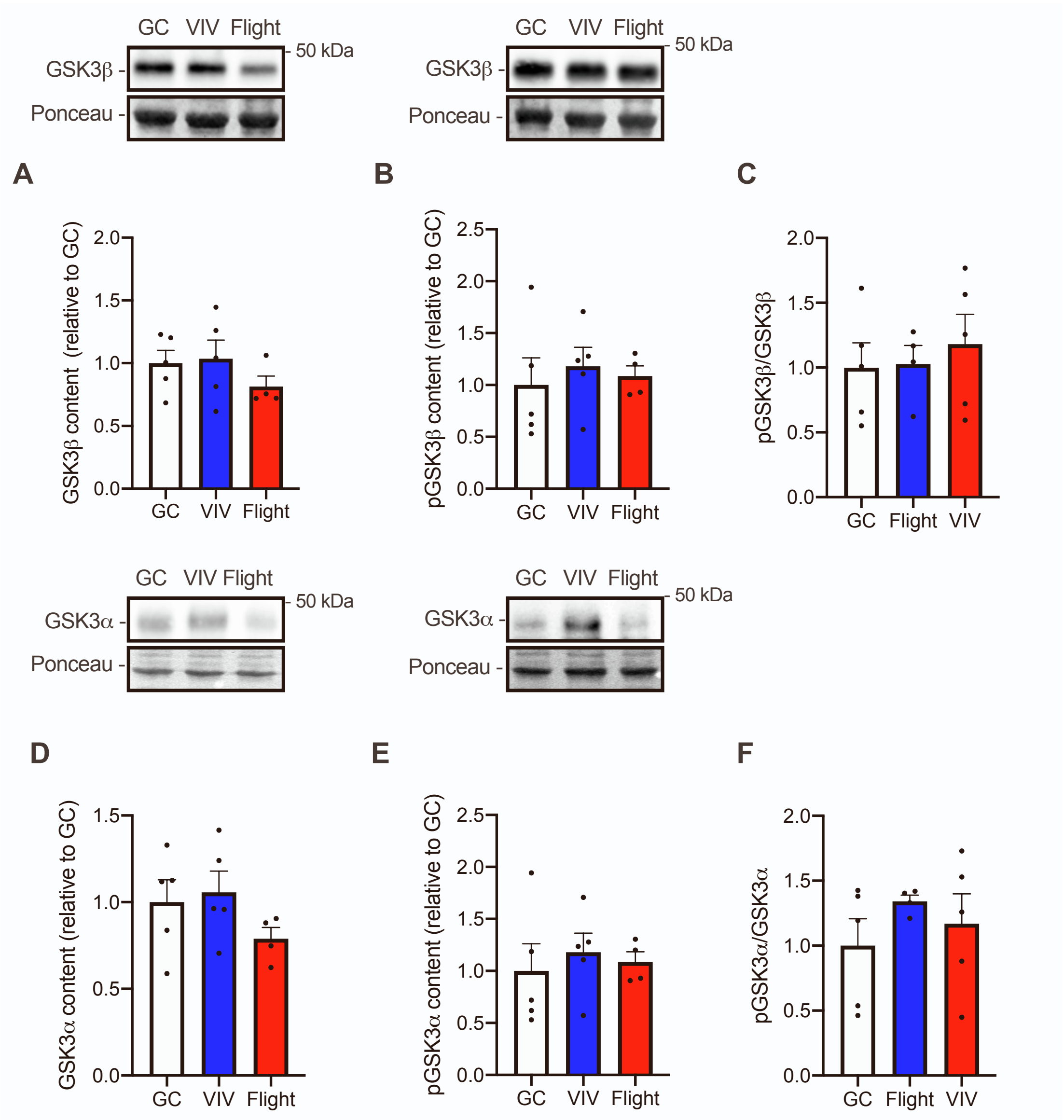


**Supplementary Fig. 7 GSK3 content and phosphorylation status in EDL muscles from GC, VIV and Flight mice from the BION-M1 mission, [Related to Figure 1]. A-C)** Total, phosphorylated, and the phosphorylated:total GSK3β ratio in Flight and control groups. **B)** Phosphorylated GSK3β (Ser9) content. **D-F)** Total, phosphorylated, and the phosphorylated:total GSK3α ratio in Flight and control groups. Data are presented as means ± SEM, n = 4-5 per group.

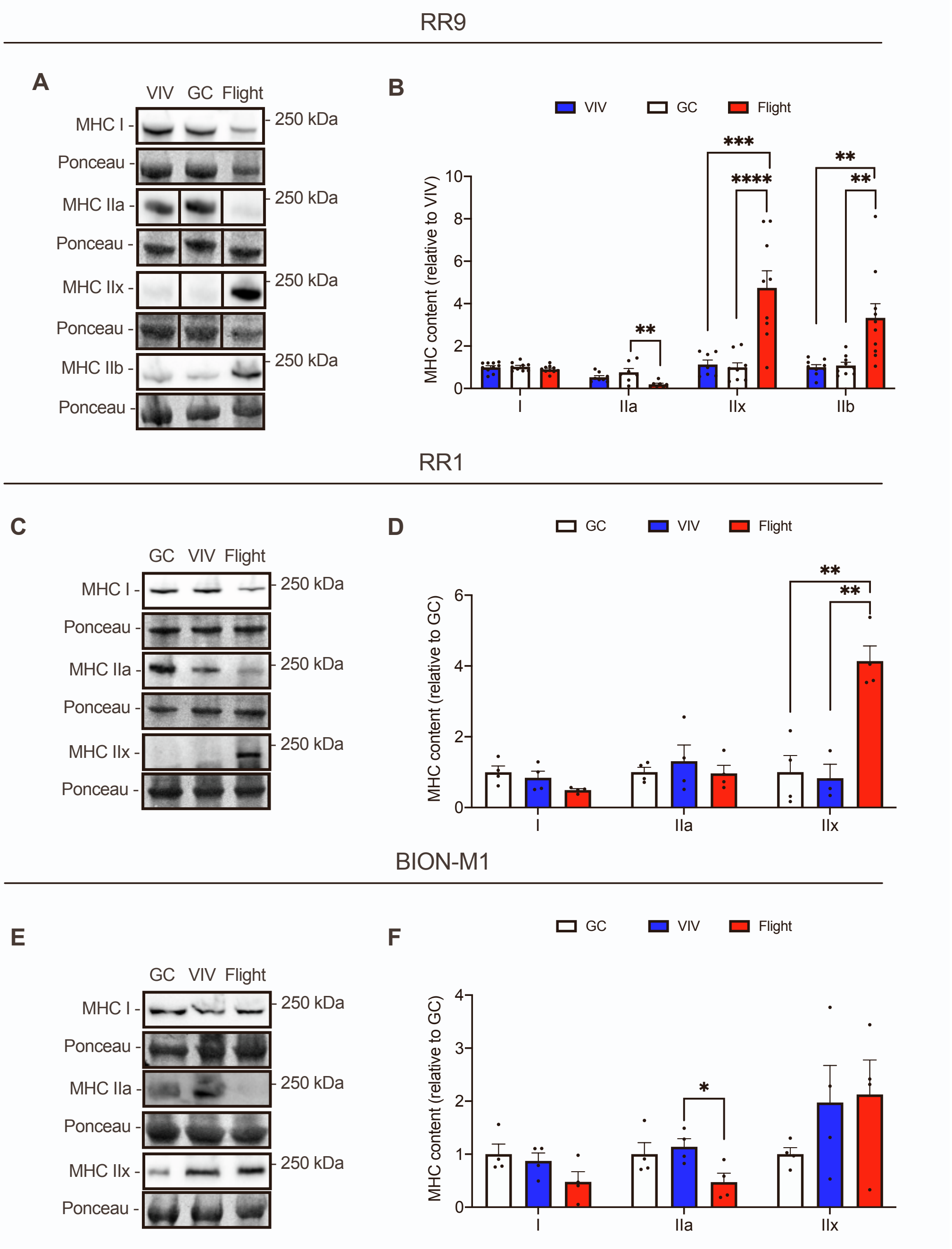


**Supplementary Fig. 8. Western blot analysis of MHC isoforms across RR9, RR1, and BION-M1 missions, [Related to Figure 1]. A-B)** MHC isoform analysis in soleus muscles from the RR9 mission (n=8-10 per group). **C-D)** MHC isoform analysis in soleus muscles from the RR1 mission (n=3-4 per group). **E-F)** MHC isoform analysis in soleus muscles from the BION-M1 mission (n=4 per group). Data are presented as means ± SEM, *p<0.05, **p<0.01, ***p<0.001, ****p<0.0001 using a one-way ANOVA or Kruskal-Wallis (D, MHCI) with Dunnett's post-hoc test. For A, the representative blots for MHC IIa and IIx were loaded in GC, VIV, Flight on the same gel, and displayed here as VIV, GC, and Flight.

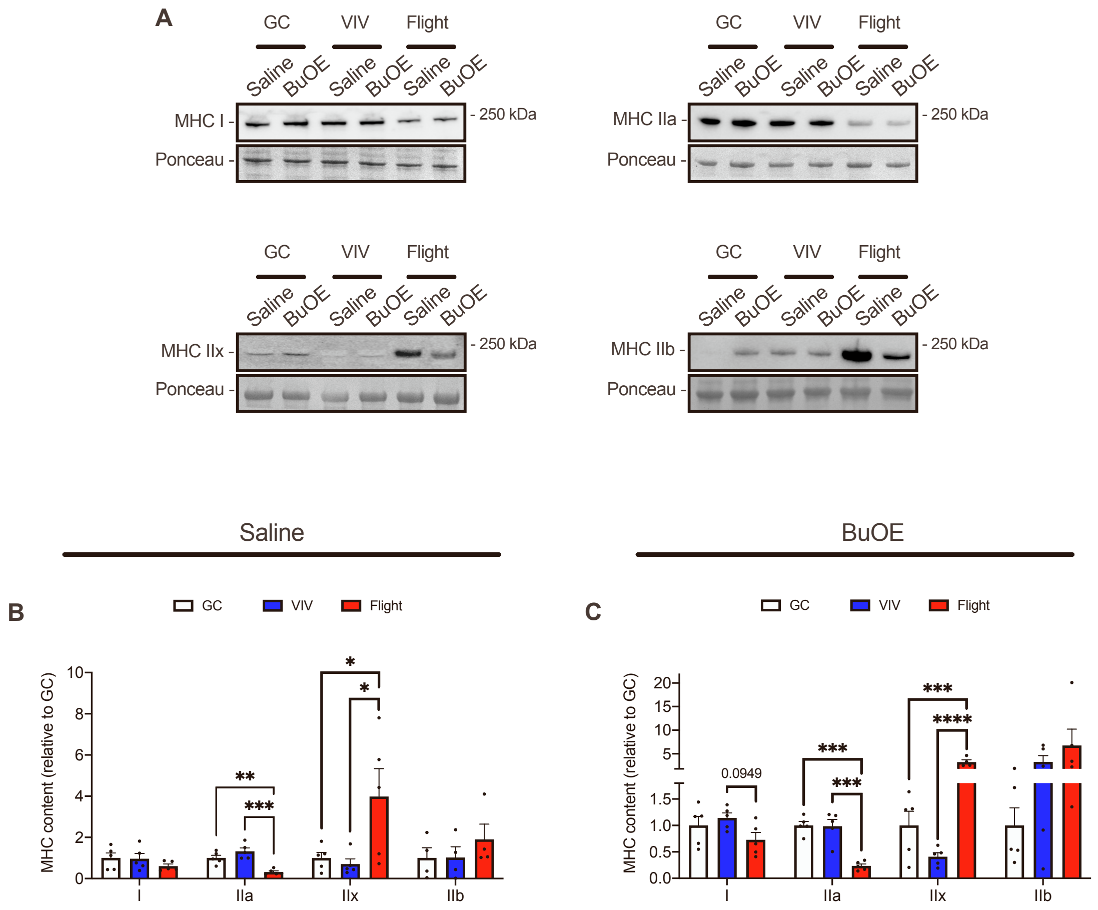


**Supplementary Fig. 9. Western blot analysis of MHC isoforms in soleus muscles from saline and BuOE-treated mice from the RR18 mission, [Related to Figure 1]. A)** Representative western blot images of MHC isoforms. **C)** MHC isoform analysis in soleus muscles from the saline-treated RR18 mice. **C)** MHC isoform analysis in soleus muscles from the BuOE-treated RR18 mice. Data are presented as means ± SEM, *p<0.05, **p<0.01, ***p<0.001, ****p<0.0001 using a one-way ANOVA or Kruskal-Wallis with Dunnett's post-hoc test (n = 4-5 per group).

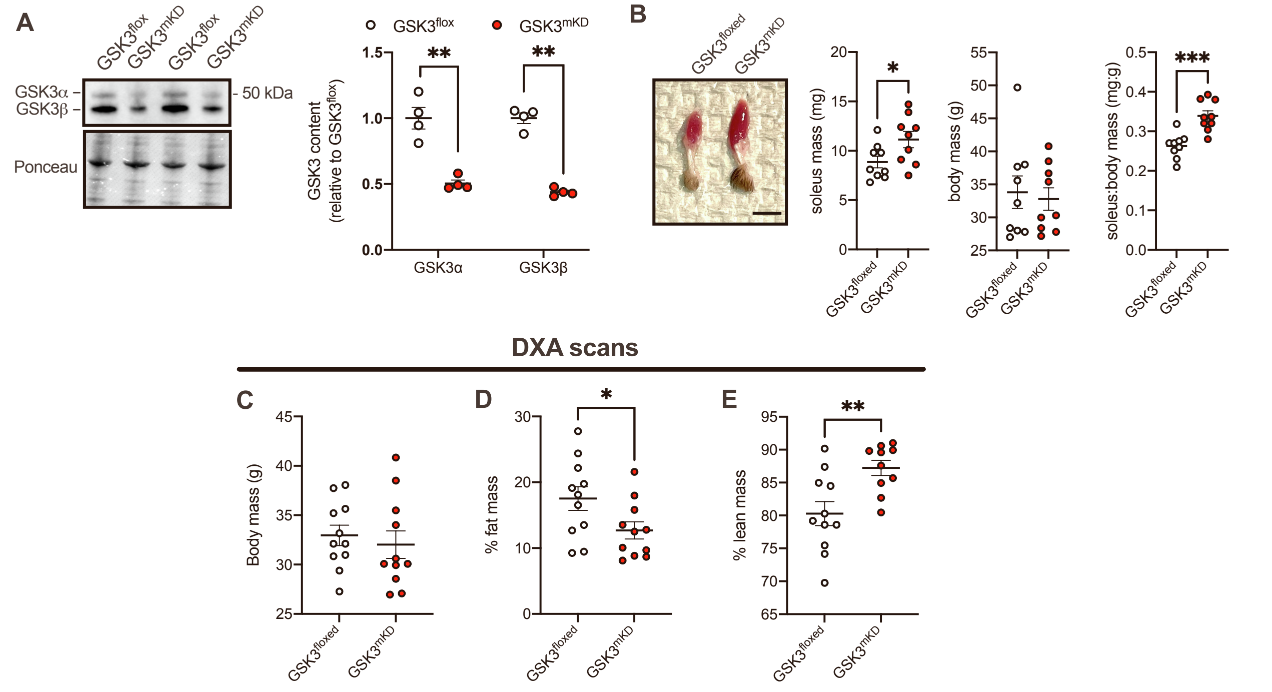


**Supplementary Fig. 10. Partial (40-50%) knockdown of GSK3 in muscle increases soleus muscle size, reduces percentage body fat, and increases percentage lean mass in male mice, [Related to Figure 3]. A)** Western blot analysis of GSK3α and GSK3β in soleus muscles from GSK3$^{mKD}$ mice and GSK3 flox (GSK3$^{flox}$) control mice (n = 4 per group). **B)** Absolute and relative (to body mass) soleus muscle mass in GSK3$^{mKD}$ mice and GSK3$^{flox}$ mice (n = 9 per group). **C-D)** DXA scan analyses showing that GSK3$^{mKD}$ mice have no change in body mass but have lowered % fat mass and increased % lean mass. Data are presented as means ± SEM, *p<0.05, **p<0.01, ***p<0.001, using a Student's t-test.

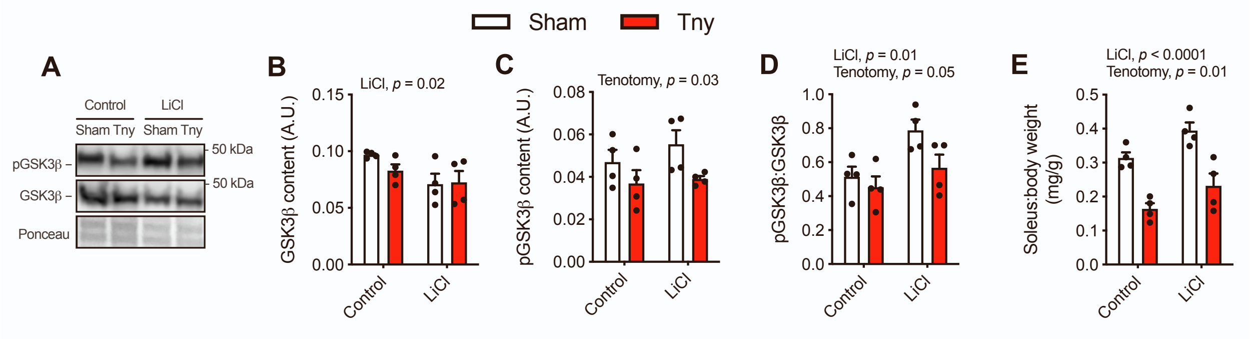


**Supplementary Fig. 11. Low dose lithium (10 mg/kg/day lithium chloride [LiCl]) treatment inhibits GSK3 and increases soleus:body mass ratio even in the tenotomized state, [Related to Figure 3].** Male C57BL/6 mice (3-6 months old) were fed LiCl via their drinking water or standard water (control) for a total of 6 weeks. On the 4th week, all mice were subjected to tenotomy surgery, severing the soleus and gastrocnemius tendon. After 6 weeks the mice were euthanized and soleus muscles were collected. **(A)** Representative western blot images of Ser9 phosphorylated and total GSK3β in the sham and tenotomized (Tny) soleus. **(B-C)** Quantification of total GSK3β and Ser9 phosphorylated GSK3β normalized to ponceau. **(D)** Phosphorylated GSK3β:total GSK3β ratio. **(E)** Soleus:body mass ratio in sham and Tny conditions. For B-E, a two-way ANOVA testing the main effects of LiCl and tenotomy. Significant main effects are denoted in each bar graph. All data are expressed as means ± SEM.

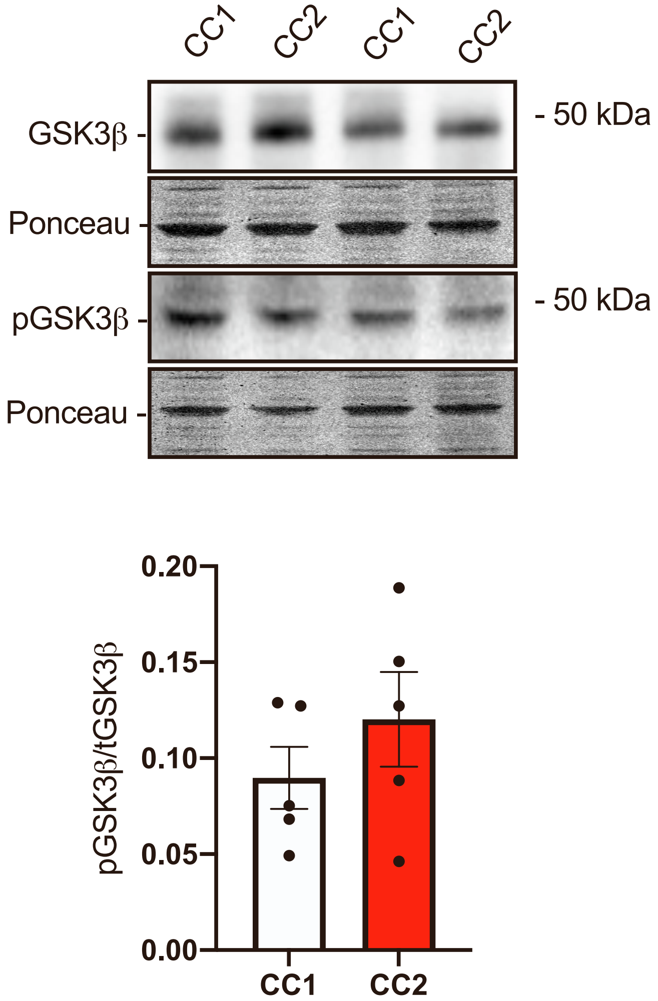


**Supplementary Fig. 12 Phosphorylated GSKβ status (relative to total) in cohort control 1 (CC1) and cohort control 2 (CC2) is not statistically different (n=5 per group), [Related to Figure 1].**

**Supplementary Table 1. Soleus wet weights and body mass from the RR18 mission, [Related to Figure 1].**

| | GCVIV (mean ± SEM) | Flight (mean ± SEM) | *p*-value (t-test) |
|---|---|---|---|
| *RR18 saline* | | | |
| Soleus (mg) | 8.9 ± 0.2 | 7.1 ± 0.6 | 0.004 |
| Body mass (g) | 28.9 ± 0.7 | 27.1 ± 1.2 | 0.15 |
| Soleus:body mass | 0.31 ± 0.01 | 0.26 ± 0.02 | 0.04 |
| | | | |
| *RR18 BuOE* | | | |
| Soleus (mg) | 9.1 ± 0.5 | 7.1 ± 0.4 | 0.02 |
| Body mass (g) | 28.8 ± 0.5 | 28.1 ± 0.7 | 0.41 |
| Soleus:body mass | 0.32 ± 0.01 | 0.25 ± 0.01 | 0.02 |

GCVIV n = 9-10, Flight n = 5

**Supplementary Table 2. Tibia structural analysis measured using μCT in the Flight and combined GCVIV control group, [Related to Figure 4].**

| | **GCVIV (mean ± SEM)** | **Flight (mean ± SEM)** | ***p*-value (t-test)** |
|---|---|---|---|
| *Trabecular bone at proximal tibia* | | | |
| Total volume ($mm^3$) | 1.4 ± 0.04 | 1.3 ± 0.04 | 0.20 |
| Bone volume ($mm^3$) | 0.23 ± 0.02 | 0.17 ± 0.01 | 0.12 |
| Bone volume fraction (%) | 16.5 ± 1.1 | 13.5 ± 1.1 | 0.17 |
| Trabecular thickness (mm) | 0.053 ± 0.003 | 0.048 ± 0.003 | 0.23 |
| Trabecular separation (mm) | 0.19 ± 0.004 | 0.20 ± 0.003 | 0.27 |
| Trabecular number (1/mm) | 3.0 ± 0.1 | 2.8 ± 0.1 | 0.30 |
| Degree of anisotropy | 2.1 ± 0.05 | 1.9 ± 0.06 | 0.21 |
| Connectivity density (1/$mm^3$) | 0.0003 ± 0.00001 | 0.0004 ± 0.00003 | 0.14 |
| | | | |
| *Cortical bone at tibia midpoint* | | | |
| Total cross-sectional area ($mm^2$) | 1.4 ± 0.03 | 1.2 ± 0.02 | 0.005 |
| Cortical area ($mm^2$) | 0.8 ± 0.03 | 0.6 ± 0.01 | 0.003 |
| Cortical area fraction (%) | 57.4 ± 0.8 | 53.7 ± 0.7 | 0.01 |
| Periosteal perimeter (mm) | 7.8 ± 0.1 | 7.3 ± 0.1 | 0.01 |
| Total perimeter (mm) | 4.9 ± 0.1 | 4.5 ± 0.0 | 0.005 |
| Endocortical perimeter (mm) | 2.9 ± 0.1 | 2.8 ± 0.0 | 0.09 |
| Medullary area ($mm^2$) | 0.61 ± 0.01 | 0.55 ± 0.01 | 0.24 |
| Eccentricity | 0.67 ± 0.01 | 0.64 ± 0.00 | 0.19 |
| Cortical thickness (mm) | 0.21 ± 0.00 | 0.18 ± 0.00 | 0.01 |

μCT, micro-computed tomography. GCVIV n = 10, Flight n = 4.

**Supplementary Table 3. Femur BMC, BMD, and tibia structure measured using DXA and μCT in RR9 cohort controls 1 and 2 (CC1 and CC2), [Related to Figure 4].**

| | **CC1 (mean ± SEM)** | **CC2 (mean ± SEM)** | ***p*-value (t-test)** |
|---|---|---|---|
| *Whole Femur* | | | |
| BMC (mg) | 24.2 ± 1.2 | 24.8 ± 1.9 | 0.08 |
| BMD ($mg/cm^2$) | 81.6 ± 2.2 | 86.7 ± 1.5 | 0.80 |
| | | | |
| *Trabecular outcomes of tibia* | | | |
| Total volume ($mm^3$) | 1.2 ± 0.03 | 1.3 ± 0.02 | 0.35 |
| Bone volume ($mm^3$) | 0.16 ± 0.01 | 0.19 ± 0.01 | 0.14 |
| Bone volume fraction (%) | 13.0 ± 1.0 | 15.4 ± 1.4 | 0.19 |
| Trabecular thickness (mm) | 0.051 ± 0.002 | 0.052 ± 0.003 | 0.62 |
| Trabecular separation (mm) | 0.22 ± 0.006 | 0.20 ± 0.006 | 0.07 |
| Trabecular number (1/mm) | 2.6 ± 0.1 | 2.9 ± 0.2 | 0.10 |
| Degree of anisotropy | 1.8 ± 0.04 | 2.0 ± 0.05 | 0.05 |
| Connectivity density ($1/mm^3$) | 0.0003 ± 0.00002 | 0.0004 ± 0.00003 | 0.09 |
| | | | |
| *Cortical outcomes of tibia* | | | |
| Total cross-sectional area ($mm^2$) | 1.3 ± 0.05 | 1.3 ± 0.05 | 0.91 |
| Cortical area ($mm^2$) | 0.7 ± 0.02 | 0.8 ± 0.02 | 0.74 |
| Cortical area fraction (%) | 55.4 ± 1.3 | 56.5 ± 0.2 | 0.41 |
| Periosteal perimeter (mm) | 7.5 ± 0.2 | 7.5 ± 0.1 | 0.83 |
| Total perimeter (mm) | 4.7 ± 0.1 | 4.7 ± 0.1 | 0.88 |
| Endocortical perimeter (mm) | 2.9 ± 0.1 | 2.8 ± 0.0 | 0.52 |
| Medullary area ($mm^2$) | 0.60 ± 0.03 | 0.58 ± 0.02 | 0.64 |
| Eccentricity | 0.68 ± 0.02 | 0.70 ± 0.01 | 0.40 |
| Cortical thickness (mm) | 0.20 ± 0.00 | 0.20 ± 0.00 | 0.46 |

μCT, micro-computed tomography. n = 5 per group.
